# Farming of Indigenous Crayfish in Russia: A Mini-Review of Recent Studies

**DOI:** 10.3390/ani15020223

**Published:** 2025-01-15

**Authors:** Alexander G. Dvoretsky, Vladimir G. Dvoretsky

**Affiliations:** Murmansk Marine Biological Institute of the Russian Academy of Sciences (MMBI RAS), 183038 Murmansk, Russia; ag-dvoretsky@yandex.ru

**Keywords:** noble crayfish, narrow-clawed crayfish, *Astacus astacus*, *Pontastacus leptodactylus*, *Pontastacus cubanicus*, Russia, aquaculture

## Abstract

Natural populations of crayfish in Europe have experienced significant declines due to the spread of crayfish plague, overfishing, competition with invasive crayfish species, and habitat degradation. Consequently, crayfish farming has gained importance in meeting the increasing demand for crayfish products. Although Russia boasts abundant water resources, the development of crayfish aquaculture remains nascent. In this review, we consolidate recent studies by Russian researchers that focus on various aspects of crayfish cultivation. Extensive cultivation in open ponds is the most suitable method, owing to its accessibility and lower costs; however, it necessitates suitable environmental conditions within the water bodies. The production cycle encompasses pond preparation, stocking, exploration, restoration, and monitoring. Controlled cultivation of egg-bearing females is essential for producing high-quality seedstock for subsequent rearing. Experimental evidence suggests that crayfish exhibit high adaptability to artificial conditions, with formulated diets comprising artificial feeds for sturgeon and powdered dried beef by-products demonstrating promising results in terms of growth performance. Plastic tangled threads and mink shelters are recommended to reduce cannibalism. Short-term grow-out of juvenile crayfish can yield a profit margin of 17%, while a complete two-year production cycle is projected to achieve financial break-even over three years. The primary challenges confronting the industry include disease management, cannibalism, the need for skilled technical personnel, and insufficient financial and policy support.

## Simple Summary

Freshwater crayfish are important organisms in terms of their ecological role and reputation as a delicacy and food source with cultural significance. In Russia, crayfish are abundant in water bodies and are extensively harvested. In this review, we summarize recent information on the biology of the main species, as well as aquaculture practices which are being developed to meet the growing demand for high-quality products. We discuss the main problems in the industry and provide further directions for the development of crayfish farming in Russia.

## 1. Introduction

Freshwater crayfish, classified within the family Astacidae Latreille, 1802, represent a notably successful group of decapod crustaceans [1]. This group, comprising over 500 species, has achieved a near-global distribution, often aided by anthropogenic introductions. These organisms are esteemed not only for their prolific status as a food resource but also for their unique morphological characteristics, which have rendered them culturally significant across numerous societies. This significance is evidenced in various cultural expressions, including culinary traditions, artistic depictions, and folklore [1]. Beyond their harvest from wild populations in fisheries, several crayfish species have been domesticated and cultivated intensively for aquacultural production [1]. Historically, crayfish have exerted substantial sociocultural influence throughout Europe since the Medieval period [2]. Larger crayfish species, particularly the noble crayfish, were disseminated by monastic and royal entities, thereby becoming integral to the diets of both the general populace and nobility. Presently, crayfish-centric traditions endure, with annual festivities such as harvest celebrations prominently observed in regions including the Pacific Northwest and Scandinavia [3,4]. Moreover, various crayfish components, especially gastroliths, have been utilized in traditional medicine practices for centuries, further illustrating their cultural and economic significance [1].

Despite their historical and cultural prominence, many freshwater crayfish species face significant threats. Their limited capacity for dispersal into alternate aquatic environments, in response to deteriorating habitat conditions, often results in population declines or extinction events [5]. As the largest motile invertebrates inhabiting freshwater ecosystems, crayfish frequently fulfill the role of “keystone species”, defined as organisms whose effects on their community or ecosystem are disproportionately large relative to their abundance [6]. This designation underscores their substantial ecological significance. Furthermore, crayfish are recognized as “umbrella species”, which are characterized by rigorous habitat and spatial requirements; conserving such species is expected to facilitate the preservation of numerous other organisms that share their habitat [6]. Notably, crayfish also provide essential habitat for a variety of smaller ectosymbiotic organisms that inhabit their exoskeletons [7,8,9]. The decline of crayfish populations in natural settings can precipitate adverse ecological cascades, negatively impacting regional ecosystems and biodiversity [10]. Anthropogenic activities, including river damming, fish stocking for recreation, and urban development, have adversely affected freshwater ecosystems, leading to both the extinction of local crayfish populations and the emergence of new, endangered species [11,12].

Russia boasts an impressive marine water profile, with a coastline stretching over 37,653 km along the Pacific, Arctic, and Atlantic Oceans, as well as the Caspian and Black Seas. The country’s exclusive economic zone covers approximately 7.6 million km^2^, offering access to significant marine resources, including fish stocks, oil, and gas reserves [13,14,15]. Russia is also notably advanced in terms of its extensive water resources, comprising over 2.5 million rivers and streams. Furthermore, Russia is home to approximately 2.7 million lakes, covering an area of 409,000 km^2^, including 11 major lakes that each span over 1000 km^2^. This abundance of freshwater bodies underscores Russia’s rich diversity in both natural and anthropogenic aquatic ecosystems [16]. According to Borisov [17], the native freshwater fauna also includes 30 species of benthic crustaceans, among which are the following seven species of crayfish: *Astacus astacus* (L., 1758), *Pontastacus leptodactylus* (Eschscholtz, 1823), *Pontastacus angulosus* (Rathke, 1837), *Pontastacus salinus* (Nordmann, 1842), *Pontastacus cubanicus* (Birstein and Winogradow, 1934), *Pontastacus eichwaldi* (Bott, 1950), and *Caspiastacus pachipus* (Rathke, 1837).

During the late 19th and early 20th centuries, Russia and the Soviet Union emerged as leaders in global crayfish production, attributable to their abundant populations and expansive aquatic habitats suitable for crayfisheries. This region accounted for over 50% of the industry’s total output, which was approximately 2000 tons annually [16,18]. This industrial success was largely based on the exploitation of crayfish resources from natural waters in regions such as the Northwest, Central Volga, and the North Caucasus [18,19,20,21]. In the 1970s, however, the dynamics of crayfish production changed dramatically due to the unintentional introduction of crayfish plague, a virulent pathogen that devastated crayfish populations [1]. This crisis was exacerbated by additional environmental pressures, such as eutrophication and the pollution of lakes and rivers. By the beginning of the 21st century, according to Alexandrova [18], the crayfish harvest had decreased by 11 times compared to the earlier part of the century. The industry was geographically confined to the Don, Kuban, and Volga river basins, reflecting a significant reduction in both the size and scope of crayfish fisheries in the country. Currently, annual catches are relatively stable, ranging from 250 to 275 tons in recent years [16]. The main fishing areas for crayfish in Russia include the Volgograd Reservoir, the Saratov Reservoir, the water bodies of the left bank of the Volga River, and the Kuibyshev Reservoir [16].

Crayfish have historically been utilized for a variety of purposes, including as a food source, fish bait, research specimens, educational tools, pets, and objects of cultural significance [1]. The earliest documented efforts in crayfish cultivation in Russia trace back to the 19th century, where Siberian merchants and manufacturers raised crayfish in rainwater barrels, which were insulated with towels. Over time, crayfish emerged as popular delicacies appreciated by both the nobility and the general populace. The deliberate commercial culture of crayfish appears to have originated in the 1930s and 1940s, with literature from that period documenting their use for both fish bait and culinary purposes [22]. Crayfish farming is regarded as a potentially profitable enterprise, given the elevated market price and the demand for the product both locally and for export [22]. Extensive or semi-extensive production approaches are typical of commercial crayfish aquaculture [23]. Prior to the 1990s, the farming of freshwater crayfish was primarily conducted in the southern states of the USA, Australia, and Europe. Concurrently, at least equivalent amounts were harvested from the wild in North America, China, Australia, Kenya, Turkey, and Europe [23,24,25,26]. Over the past few decades, international trade in crayfish has significantly expanded, largely due to the rise in farm production in China [22].

The regions of Russia possess considerable potential for the development of aquaculture [27], particularly in the production of high-value seafood species such as salmon, sturgeon, and shellfish [28,29,30]. The country’s extensive coastline offers a diverse array of habitats, which are suitable for various types of aquaculture systems, including fish and shellfish farming, seaweed cultivation, and integrated multi-trophic aquaculture systems. In addition, despite the imperative to conserve natural crayfish populations, crayfish farming in Russia remains underdeveloped on an industrial scale. For example, during the period from 2012 to 2023, data pertaining to the cultivation of crayfish are available specifically for the years 2015 to 2018, during which total annual production fluctuated between 2 and 4 tons. This is surprising, given the country’s favorable resources, which include endemic crayfish species of significant consumer value [18], abundant water bodies amenable to the establishment of crayfish farming operations, and a wealth of methodological innovations developed by research institutes of the former USSR [31,32,33,34,35,36]. Unfortunately, much of this valuable research is published in Russian, limiting its accessibility to the international scientific community.

The aim of our paper is to synthesize the available information concerning the biological characteristics and current population status of native crayfish species, as well as to explore the aquaculture techniques applicable to these organisms. By collating and analyzing existing data and research, this work seeks to provide a comprehensive understanding of crayfish biology and outline sustainable practices for enhancing crayfish aquaculture in Russia.

## 2. Biological Aspects

### 2.1. Noble Crayfish

The noble crayfish (*Astacus astacus*) inhabits a diverse range of freshwater environments, such as streams, rivers, lakes, ponds, and reservoirs. These habitats, which can be located in both lowland and hilly regions, exhibit considerable variability in factors like substrate composition, flow patterns, aquatic vegetation, and levels of organic enrichment. For optimal living conditions, the species requires suitable shelter, including stones, wood, logs, roots, and vegetation, commonly available in these natural settings [16]. While the noble crayfish prefers making simple burrows in clay and earthen banks, it can also thrive in earthen culture ponds, though it struggles in pools with muddy bottoms. The species can tolerate some degree of organic enrichment, but its prevalence in nutrient-rich waters is constrained by low oxygen levels, contamination from toxic substances, and the sediment associated with eutrophication [37].

For successful reproduction, water temperatures of at least 15 °C during the summer months are generally thought to be necessary, although reproduction may still occur at lower temperatures in running waters [16]. Optimal water temperatures for growth range between 16 and 24 °C, with higher temperatures up to 28 °C being tolerable only briefly [38]. Oxygen concentrations need to be at least 3 to 4 mg/L for survival, with higher concentrations essential for successful brood development. If oxygen levels fall below these thresholds, the crayfish may attempt to leave the water, if possible [37].

The distribution of the noble crayfish is heavily influenced by the composition of the benthic substrate—the material found at the bottom of aquatic environments [39]. When not feeding, crayfish typically hide in burrows or seek cover under stones, logs, vegetation, roots, and leaf litter. Larger crayfish tend to choose larger stones and deeper burrows for concealment. The presence of predatory fish can limit the habitat use and activity of crayfish. Furthermore, the location and type of habitat affect the size composition of the crayfish population [37].

The exoskeleton of crayfish imposes limitations on their continuous growth in both length and weight, necessitating periodic molting, a phenomenon also observed in other crustacean species. In *Astacus astacus*, males typically undergo the molting process twice a year, contingent upon favorable temperature conditions and the availability of food resources. The initial molt generally occurs between mid-spring and early summer, followed by a second molt from late summer to early autumn. In contrast, females, preoccupied with the care of their juveniles, typically molt only once per year, which occurs in late summer or early autumn [37].

Noble crayfish exhibit a varied and adaptable diet that reflects their habitat conditions, functioning as omnivores, predators, or detritivores. Their dietary components encompass detritus, algae, invertebrates, vertebrates, fish and fish eggs, aquatic vascular plants, carrion, and periphyton [40]. Adult crayfish are subjected to predation by various fish species, including eels (*Anguilla anguilla*), perch (*Perca fluviatilis*), northern pike (*Esox lucius*), and brown trout (*Salmo trutta*). Additionally, avian predation comes from herons (Ardea), egrets (Ciconiiformes), seagulls (Laridae), ducks (Somateria), and loons (Gaviidae) [37].

The noble crayfish is relatively sensitive to pollution and physical disturbances in its environment [41]. Like other crayfish species, its habitat use is likely influenced by a combination of factors, including predation risk, competition with other crayfish, and temperature [26]. This crayfish species is not typically territorial. However, in confined environments where space is limited, they may exhibit aggressive behavior [16].

**Figure 1:**
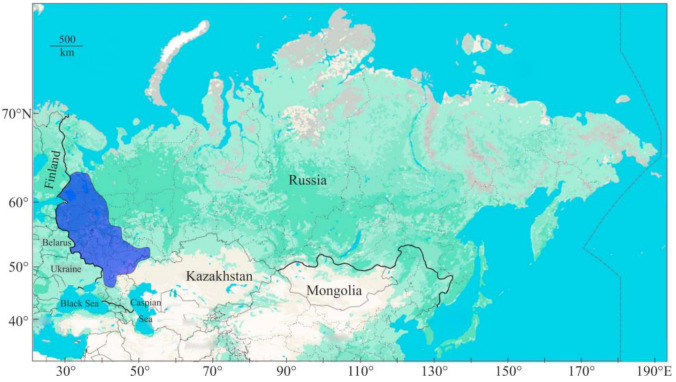
Distribution area of noble crayfish in Russia (modified from [42]).

This species is distributed in the north-western and central parts of Russia (Figure 1).

The sex ratio of noble crayfish in Russian aquatic environments approaches parity, generally manifesting as approximately 1:1 [16]. Fecundity for this species ranges from 132 to 194 eggs [16], while the maximum carapace length is reported to be approximately 14 mm [32]. The yield of edible meat is estimated to constitute 17.6% of the total body weight [32].

### 2.2. Narrow-Clawed Crayfish

The natural distribution of the narrow-clawed crayfish is primarily confined to Eastern European countries. In several regions, this species has been introduced to artificial freshwater systems such as quarry ponds and canals, where it often establishes thriving populations [16].

**Figure 2:**
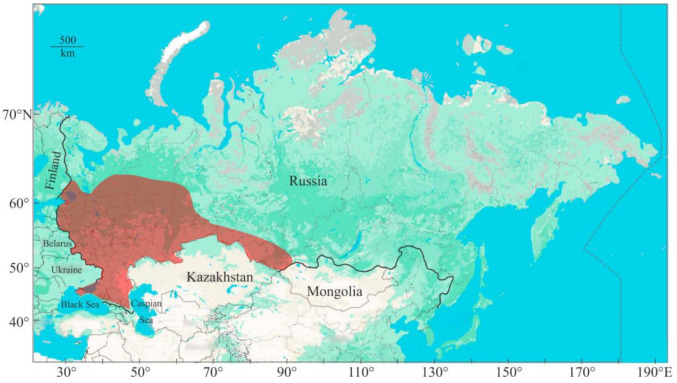
Distribution area of narrow-clawed crayfish in Russia (updated from [42]).

Narrow-clawed crayfish exhibit a broad range of habitat preferences, inhabiting a wide range of aquatic environments, including shallow and deep lakes, rivers, and streams, and a variety of substrate types [43], with a preference for stone and wood shelters [44]. Studies by Skurdal and Taugbøl [37] indicate that this species can be found in deep lakes reaching depths of up to 50 m, and even in brackish water. Unlike the noble crayfish, the narrow-clawed crayfish is less reliant on substantial shelter due to its increased daytime activity. This species exhibits greater resistance to pollution and wider temperature tolerance compared to other indigenous European crayfish. It is also documented in the colder climates of Western Siberia (Figure 2), highlighting its ability to withstand a variety of environmental conditions [16].

The narrow-clawed crayfish can adapt to numerous bottom substrate types, except those heavily silted, and can endure a wide range of temperature fluctuations (4–32 °C), salinity changes (4–14 psu), and temporary decreases in oxygen concentration to as low as 2 mg L^−1^ [45]. Consequently, populations of this species inhabit both brackish and freshwater environments, including lagoons and estuaries in the Caspian Sea, Azov Sea, and Black Sea, as well as the open sea off the eastern Caspian coast [16].

Narrow-clawed crayfish are omnivorous organisms that feed on a diverse array of food sources. For the plant-based component of their diet, they consume various aquatic plants and submerged macrophytes, including genera such as *Ceratophyllum*, *Elodea*, and *Potamogeton*, as well as Charophyceae. The latter group constitutes 10–40% of the diet, while Elodea accounts for approximately 6%. Noble crayfish also ingest components of the reed ecosystem and members of the Cyperaceae family, which comprise about 2.5% of their dietary intake [46]. The animal-based portion of their diet includes oligochaetes, mollusks, amphipods, insect larvae, tadpoles, frogs, and small fish. Crayfish larvae primarily feed on daphnia (59%), chironomids (25%), and plant matter (16%). Juvenile crayfish are known to consume chironomids, which comprise 24–25% of their diet. Specimens that are one year old and measure approximately 2 cm in body length begin to include insects and larval insects in their diet, and these contribute 18–45%. Additionally, in crayfish measuring 8–10 cm, amphipods can constitute up to 63% of the dietary intake [46]. Predation pressure on narrow-clawed crayfish primarily comes from fish (including pike, perch, and pikeperch), as well as from avian predators such as storks, and terrestrial predators like water voles and foxes.

The age at sexual maturity for narrow-clawed crayfish is approximately 3 years for males and 4 years for females. In Russian aquatic systems, mating occurs during the spring months. During copulation, the male deposits spermatophores on the ventral surface of the female. Egg hatching commences 3–4 weeks following fertilization [46]; the fertilized eggs subsequently adhere to the setose pleopods located beneath the female’s abdomen [45]. The incubation period continues until the subsequent June or July, necessitating constant aeration, which the female facilitates through the rhythmic bending and unbending of her tail, to ensure optimal egg development. Post-hatching, the larvae cluster beneath the female. Following the first molting event, the larvae begin to disperse in search of shelter within their immediate environment and ultimately detach from the female after the second molt, typically occurring at approximately 1.5–2 months of age [46].

This species shows remarkable tolerance to different substrate types, from hard and rocky to soft and muddy. Among indigenous species, it has the highest tolerance to challenging environmental conditions such as low oxygen levels, reduced water transparency, and temperature variations. Souty-Grosset et al. [2] provided examples of this resilience, noting the species’ ability to survive in waters with oxygen concentrations as low as 0.5 mg L^−1^ at a resting metabolic rate, withstand optimal temperatures ranging from 20–30 °C without lethal effects, and adapt to rapid temperature changes from 26 °C down to 15 °C and up to 35.8 °C. The sex ratio of the narrow-clawed crayfish typically averages 1:1, although it can exhibit variability ranging from 3:1 to 1:5 [16]. The average fecundity of the narrow-clawed crayfish varies considerably, spanning from 117 to 523 eggs, a variation influenced by local thermal regimes, with lower fecundity observed at higher latitudes and/or in arid regions [16]. In terms of size, maximum lengths range from 13 cm in the central regions of Russia [33] to 22 cm in Siberian habitats [47]. The meat yield of this species is reported to range from 18.9% to 27.8% of body mass [16].

Traditionally, the narrow-clawed crayfish was considered a complex species comprising three subspecies: *Pontastacus leptodactylus leptodactylus* and *Pontastacus leptodactylus cubanicus* in freshwater sources and *Pontastacus leptodactylus eichwaldi* in brackish waters. These are now regarded as separate species [48].

## 3. Aquaculture Techniques

### 3.1. Cultivation Approaches and Requirements

Various techniques can be used to increase crayfish yields, including stewardship of wild populations, extensive production in natural or artificial ponds, semi-intensive cultivation in ponds and raceways, intensive production in tanks, basins, or raceways, and systems involving the rotational cultivation of crayfish with plants [18].

A specific method for the extensive cultivation of crayfish has been developed to accommodate the conditions of mixed forests in the European part of Russia. The successful development of crayfish aquaculture in open ponds is fundamentally contingent on the suitability and quality of water for economic utilization [18]. The criteria for appropriate water bodies for crayfish farming include high dissolved oxygen and calcium levels, low alkalinity and iron ion content, minimal organic matter, consistent water temperature, warm hypolimnetic water, low silt content in sediments, and the presence of shelters and adequate food sources (Table 1).

Furthermore, the water should be free from harmful biotic and abiotic components, such as the oomycete *Aphanomyces astaci*, and pollutants. The presence of predatory fish such as perch and walleye and foraging competitors such as cyprinids, non-native crayfish, and vertebrates including ducks, muskrats, and otters is also not advisable [36].

**Figure 3:**
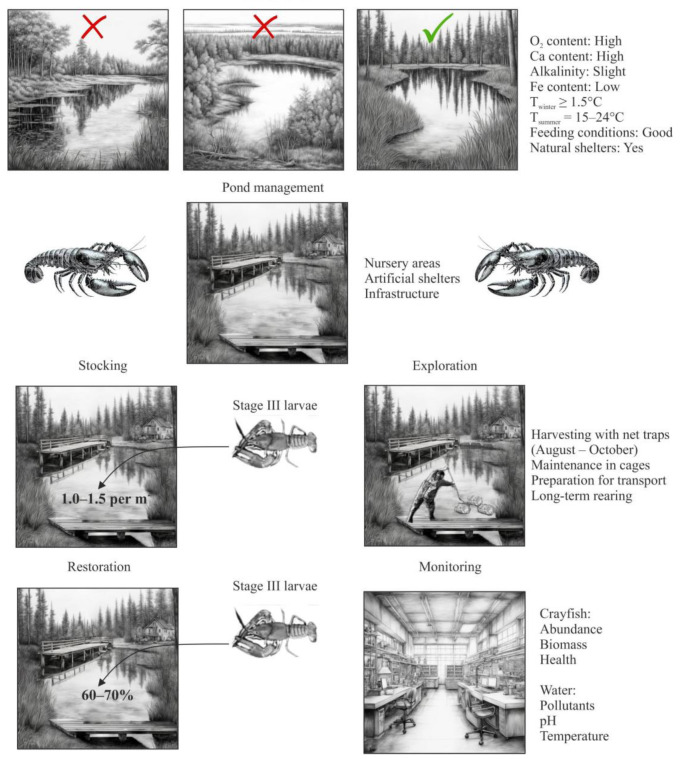
Production cycle for extensive aquaculture of crayfish (adopted from [31]).

The aquaculture production cycle for crayfish encompasses several key stages: pond preparation, stocking, exploration, restoration, and monitoring (Figure 3).

Pond preparation typically requires 2–3 years to establish optimal productivity conditions, with an initial stocking density of 2600 individuals per hectare to ensure a yield of at least 975 market-sized crayfish per hectare and meat yields exceeding 19% for noble crayfish (*Astacus astacus*) and 16–17% for narrow-clawed crayfish (*Pontastacus leptodactylus*). A sustainable annual harvesting rate of 25–30% of the commercially available biomass is maintained, significantly higher than the total allowable catch limits for wild populations [31]. Restoration involves both the natural reproduction of the retained breeding stock as well as supplemental stocking with juvenile crayfish from dedicated hatchery facilities, with the hatchery contribution accounting for 60–70% of total annual recruitment. Regular water quality monitoring and analysis is essential (Table 2), along with health screening to assess the prevalence of economically important diseases such as crayfish plague and ectosymbiont infestations by the Branchiobdellida species. In a well-managed pond, less than 5–10% of the population should show clinical signs of disease. Seedstock production is a critical step in crayfish aquaculture as it provides juveniles for grow-out in production ponds [31].

Similar approaches have been developed in other countries, with promising results. In Estonia, for example, the total annual production of market-size crayfish has reached 1000 kg, in addition to small crayfish that are sold to other crayfish farmers or used for restocking [24]. According to recent data from the Food and Agriculture Organization [49], the total annual aquaculture production of *Astacus astacus* in Bulgaria was 200 kg in 2022. In contrast, Denmark and Romania reported production figures of 90 kg and 44 kg of crayfish, respectively, in 2019. The highest recorded production of narrow-clawed crayfish during 2020 and 2021 occurred in Iran, with outputs of 13 tons and 20 tons, respectively. This was followed by Bulgaria, with production levels of 6.7 tons in 2020 and 6.6 tons in 2021, and Moldova, which maintained a consistent production of 5 tons annually during these years [49].

### 3.2. Seedstock Production

There are two main approaches for obtaining crayfish seedstock: artificial incubation of embryos removed from the female’s pleopods, or controlled cultivation of ovigerous (egg-bearing) females [50]. Artificial incubation allows multiple hatching cycles from a fixed number of eggs held in refrigerated storage, enabling large-scale seedstock production in a small facility. However, this approach is labor-intensive and requires skilled technical staff. Incubation of egg-bearing females offers distinct advantages, including a 30% lower larval injury rate, 5–7 times lower time and labor costs, and a high larval yield (70% for 15-day-old larvae at the III developmental stage). However, this approach necessitates much higher production areas [50]. Comparative analyses by Russian researchers have shown that the use of artificial incubation yields about 157.5 thousand larvae of *Pontastacus leptodactylus*, while the cultivation of gravid females results in a yield of about 630 thousand larvae. After 15 days of pond cultivation, the average stocking density reached 600,000 larvae per hectare [18,31]. Juvenile crayfish should be separated from their mothers from 1.5 to 2 months following the release of the larvae. Given that the larvae are primarily herbivorous, there is no necessity to monitor cannibalistic behavior during the early stages of their life cycle. Research conducted in other countries suggests additional ways to increase reproductive efficiency, including exposing broodstock to constant darkness during the mating and spawning season [51]. Furthermore, the thermal regime should also be considered, as elevated temperatures have been shown to negatively affect gametogenesis in male *Pontastacus leptodactylus* [52,53]

### 3.3. Production Cycle

A 2-year production cycle in ponds has been developed for *Pontastacus cubanicus* [46].

The first phase involves cultivation of early-juvenile-stage larvae with an initial mean weight of 34.6 mg and carapace length (CL) of 1.2 cm over 2.5–3.5 months. During this nursery phase, larvae reach an average weight of 3.34 g and 7.69 g at stocking densities of 50 and 30 individuals per m2, respectively. Growth accelerates by the end of the season, achieving a mean weight of 12.5 g. Mortality rates during the larval phase range from 10–15%. By age two, market-size crayfish reach 30–42 g in weight, depending on rearing conditions. An optimal stocking density of 5 ind. m^−2^ is recommended during the on-growing phase in production ponds to ensure high survival rates of 90–95%. This result is in good agreement with data from a recent study conducted under controlled conditions in tanks by Mazlum and Uzun [54], who reported that the growth performance and survival of newly hatched third instars of narrow-clawed crayfish showed a negative relationship with stocking density, which was higher at 10 ind. m^−2^ compared to at 50 and 100 ind. m^−2^. Another study reported similar relationships between stocking density and growth rates, with additional effects from temperature, which increased the negative effect of stocking density when increasing from 15 to 25 °C [55]. In addition, the presence of shelters has been shown to have a positive effect on both the survival and production of crayfish [56].

Total production over the 2-year cycle can reach 1,500 kg per hectare for 1-year-olds, and up to 2000 kg per hectare for 2-year-olds [31,46]. Regarding feeding protocols, crayfish juveniles feed primarily on zooplankton for approximately two weeks after becoming independent, before transitioning to benthic macroinvertebrate prey [46]. Therefore, production ponds should contain zooplankton densities of at least 3 g m^−3^ prior to the stocking of larvae. Once juveniles reach a CL of 2 cm, supplemental feeding with a high-protein (42.7%) formulated diet containing fish meal and milk powder (nutritional value of 257 kcal) is recommended for year 1 animals. For year 2 crayfish, a lower protein (29.3%) feed based on wheat bran and sunflower meal (nutritional value of 248 kcal) can be used [46]. Similar results have been reported by Iranian authors who tested the effects of different diets on the growth performance and body composition of narrow-clawed crayfish and found that optimal parameters were 30% protein and 370 kcal [57]. When testing artificial microparticle diets containing 52.2 to 59% protein, Turkish specialists found that the best growth performance was achieved at 56% protein [58].

Feed amounts should be adjusted based on crayfish age (Table 3). During the first month post-hatch, juveniles should be fed small amounts four times per day (11:00, 19:00, 01:00, and 05:00), with this reduced to three feedings per day (07:00, 17:00, and 23:00) for 2-month-olds. For two-year-old animals, feeds should be introduced twice a day at 17:00 and 23:00 [46]. Under controlled conditions, a similar optimal feeding frequency for adult crayfish, but at 12:00 and 24:00, was reported by Polish authors [56].

### 3.4. Case Study of Pond Cultivation

Cultivation experiments on *Pontastacus leptodactylus* in the Saratov Oblast region were detailed in a study by Kiyashko et al. [59]. The authors stocked a 400 m^2^ pond (maximum depth, 4.2 m) with 2795 juveniles (mean weight, 30 g; total biomass, 85 kg) in late May. After a 10-day acclimation period, supplemental feeding was initiated using low-cost fish (crucian carp, *Carassius gibelio*) as the protein source. For controlling feed consumption and retrieving unfed remnants, the authors used original devices comprising metal plates measuring 20 × 40 cm, with 10 cm metal pins welded to the upper side of the plates. The fish were threaded onto the spines, and the plates were placed into the pond. After a 4-month cultivation period, the study reported a survival rate of 82%, a mean weight gain of 22 g (representing a 73% increase), and a total weight gain of 34 kg (a 40% rise). The total feed weight used was 198 kg, indicating 5.8 kg of fish were used per 1 kg of crayfish wet weight gain. Economic analysis indicated a production profit margin of 16.8% [59]. The use of a low-cost, effective feed formulation and the growth rates achieved demonstrate the viability of commercial scale aquaculture production of this species to supply consumer food markets [59].

### 3.5. Case Studies of Cultivation in Recirculation Systems

Scientists from Saratov State Agrarian University conducted an experimental feeding trial using a recirculating aquaculture system to evaluate growth performance of narrow-clawed crayfish fed three different diets [60]. In this experiment, 30 crayfish were reared in twelve 250-liter aquariums, each containing polypropylene pipes serving as shelters. The study compared two experimental feeds, artificial feeds for sturgeon fish (SFF) and powdered dried beef remnants with bones (BRF), with a control diet (CD) based on fish mince. The crayfish were fed twice a day at a rate of 5% of the total biomass. Both experimental feeds resulted in higher weight gains, with SFF at 24.6 g and BRF at 19.2 g, compared to CD at 18.5 g. The daily food intake was highest for BRF at 11 g, compared to SFF and CD at 7 g. However, SFF exhibited a higher mortality rate of 30%, compared to BRF at 10% and CD at 20%, due to increased intraspecific aggression and cannibalism. The authors hypothesized this resulted from competition for SFF feed which swelled before consumption. These findings hold relevance for the ongoing development of cultivation regimes for crayfish [60]. In general, diets based on fish result in better growth performance indices than natural diets but can lead to higher mortality [61].

An experimental study conducted by specialists from the Azov Sea Research Fisheries Institute aimed to examine the adaptability of wild *Pontastacus cubanicus* to artificial conditions (similar to natural habitats) in recirculating systems with a temperature of 20 °C and an oxygen concentration ranging from 7.8 to 8.6 mg L^−1^ [62]. In their study, 50 specimens with an average length of 12.3 cm and an average weight of 45.8 g were reared for 39 days in 2.0 × 2.0 × 0.7 plastic tanks (0.5 m^3^ water volume) equipped with artificial plastic shelters. Observations revealed passive and non-feeding behaviors during the first 14 days, with feeding activity commencing from day 15 onwards. Initially, the crayfish were fed once a week with frozen fish provided as 4–5 cm pieces, after which they were transitioned to an artificial feed, BioMar, designed for sturgeon fish. By the end of the adaptation period, the daily ration had reached 4% of the total crayfish weight, leading to mean growth increments of 0.6 cm for body length and 4.4 g for body weight, with a survival rate of 85%. These findings, demonstrating higher growth rates and survival rates compared to natural conditions, serve as a promising basis for crayfish cultivation during winter months [62]. The effects of different diets on the growth performance and chemical composition of narrow-clawed crayfish are well described in the literature [58,63,64].

A pilot study was carried out to evaluate the growth performance of *Pontastacus leptodactylus* reared in highly mineralized hydroponic solutions (Biofloc technology) over a 3-month period [65]. In this study, crayfish were cultivated in 100-liter aquaria filled with aerated water at a flow rate of 1200 L h^−1^, maintaining a temperature of 20–22 °C. Each aquarium was equipped with PVC pipes with a diameter of 40 mm, utilized as shelters. The crayfish were fed a commercial aquafeed, BioMar Efico Sigma 811 R. No mortality occurred during the trial. The crayfish reared under control conditions (pH 7.8, mineralization level of 340 mg L^−1^) molted twice with mean growth increments of 4.3 ± 1.5 mm over 90 days. In contrast, the specimens cultured at pH 6.0 and a mineralization level of 1200 mg L^−1^ molted 4–6 times, achieving significantly higher growth rates with mean increments of 21.2 ± 2.5 mm [65]. Similarly promising results were reported by Kaya et al. [66], who also found no negative effects of this technology on the growth performance and hemolymph biochemistry of narrow-clawed crayfish over a 45 d period. These findings imply that the hydroponic Biofloc technology can be successfully replicated in commercial settings.

### 3.6. Management Plan for Crayfish Farm

**Figure 4:**
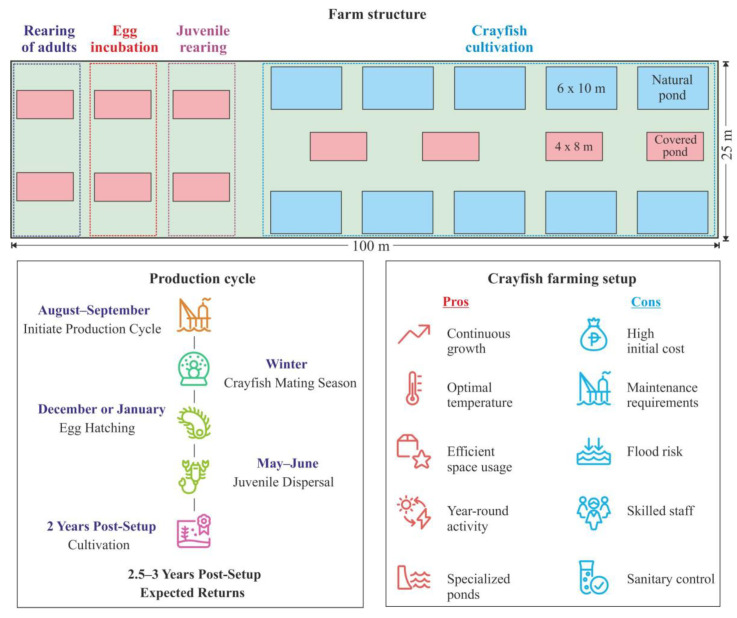
Scheme of crayfish farming (adopted from [67]).

Startseva and Kabenok [67] have elaborated a conceptual design and management plan for a *Pontastacus leptodactylus* crayfish farm tailored to the climatic conditions of the Rostov Oblast (Figure 4).

The proposed farm requires a standard land plot with an area of 2500 m^2^ (25 × 100 m), meeting specific criteria such as a flat surface not prone to regular flooding and accessibility for heavy construction machinery. The design features the installation of 20 ponds across the area, oriented from east to west and spaced at 3-m intervals. Among these, ten ponds measuring 4 × 8 m are to be covered by 8 mm polycarbonate sheeting to maintain optimal temperature conditions. This setup is intended for intensive farming, ensuring that the crayfish remain active throughout winter months, feeding and molting consistently, thus promoting continuous growth and weight gain even during the colder seasons [67]. Two ponds are allocated for the rearing of male and female crayfish, while an additional two ponds are designated for egg incubation, and a further two for the cultivation of juveniles. Moreover, the plan includes the installation of ten 6 × 10 m ponds intended for the cultivation of crayfish under natural conditions. Each of these ponds is to possess a minimum depth of 2 m, with a rectangular shape, a smooth bottom, and vertical side walls set at a 90° angle relative to the bottom. The suggested construction material for the pond structure is polypropylene sheets, chosen for their water-neutral properties and ease of connection through polyfusion welding, ensuring a sturdy and water-resistant framework. Subsequently, upon installation of the pond structure, the bottom is to be layered with 20 cm of large stones or broken ceramic bricks, followed by a 10 cm layer of coarse sand to provide shelter for the crayfish and a substrate for aquatic plants and animals. To help maintain suitable water temperatures for optimal seasonal production in the outdoor ponds, the use of a polycarbonate hoop-house or large commercial greenhouse structure over the entire system is recommended [67].

The farm should be equipped with the following essential devices: an aerator, an oxygenator, a filtration system, a pH meter, an oximeter, a thermometer, a salt meter, and a conductivity meter. The physicochemical parameters of the freshwater should include a total hardness and alkalinity of approximately 3–8 mg-equivalent L^−1^, a pH range of 7.5–8.5, oxygen levels of 6–7 mL L^−1^, and a temperature range of 22–24 °C [67].

After construction is complete, the farmer should purchase adult crayfish for both farming and establishing a breeding population. The optimal period to initiate a production cycle is August–September, prior to the spawning season. A recommended stocking density is 6 ind. m^−2^ at a sex ratio of 1 male to 3–4 females, totaling 384 crayfish (96 males and 288 females). These figures are in good agreement with those reported by Bulgarian authors, who suggested that one male can copulate with a maximum of eight females during the spawning season [68]. These individuals should be raised in two breeding ponds. In winter, when water temperatures decrease to 4–6 °C, the crayfish commence mating. During mating, the male deposits spermatophores on the ventral side of the female. Egg hatching occurs in December or January once the extruded eggs become attached to the setose pleopods underneath the tail. The incubation period persists for 6 months until the following May or June, at 14–15 °C. After hatching, the larvae aggregate under the female for 10–12 d and undergo their first molting 5–8 d after dispersing, transitioning to active feeding while remaining dependent on their parent. Following a growth period of 14–20 d, crayfish juveniles will begin to disperse from the female, seeking shelter in their immediate surroundings. Therefore, it takes approximately one month to obtain viable juveniles, with an individual female being able to carry 40–50 larvae [67]. After spawning, the females are to be separated and transferred back to the breeding ponds, while the juveniles are transferred to the cultivation ponds. For intensive cultivation, it is recommended to introduce artificial feeds to enhance growth rates [67]. The crayfish should be fed artificial diets daily, either in the morning or evening, at approximately 0.2% of their body weight. During spawning, the feeding level for females should be initiated at 0.9% of body weight at the start and reduced to 0.3% at the end of the period. Moreover, natural food items such as aquatic fauna and flora are essential additives to the artificial diet. With proper care and management, such a farm is projected to yield returns in 2.5–3 years [67].

## 4. Problems and Solutions

### 4.1. Diseases

Crayfish plague, caused by the oomycete *Aphanomyces astaci*, is among the most extensively researched diseases affecting crayfish. It is believed that this pathogen originated in North America and was introduced to Europe through human-mediated activities involving the transportation of signal crayfish *Pascifastacus leniusculus* and other species, with its initial report in Europe dating back to the 1860s [69]. While North American crayfish hosts remain asymptomatic carriers, native European Astacidea species experience high mortality rates upon exposure to *Aphanomyces astaci*. Transmission occurs via motile, waterborne zoospores released from infected moribund or deceased hosts, which can survive for several days in water or weeks in mud. Outside of its host, *Aphanomyces astaci* exists in the form of motile zoospores, which can remain viable for up to three days and subsequently form cysts capable of withstanding conditions in distilled water for up to two weeks. The maximum lifespan of *A. astaci* outside a host could extend to several weeks. Field observations indicate that outbreaks of infection caused by *A. astaci* occur within a temperature range of 4–20 °C. The optimal pH range for zoospore motility is reported to be between pH 6.0 and 7.5, with a tolerance range extending from pH 4.5 to 9.0 [70]. Furthermore, CaCl_2_ has been found to stimulate the emergence of zoospores from primary cysts, whereas MgCl_2_ exerts an inhibitory effect on this process [71]. Zoospores exhibit chemotactic attraction to crayfish hosts, adhere to and penetrate the cuticle, encyst, and form germ tubes allowing hyphal invasion of host tissues. Extensive hyphal colonization and proliferation in susceptible hosts leads to zoosporangia development and the subsequent release of infectious zoospores immediately preceding or upon host mortality [72]. The presence of damaged exoskeletons can accentuate susceptibility transmission risks. Clinical manifestation of the infection is relatively nonspecific, signified chiefly by muscle whitening, though this symptom may represent other pathological conditions [1].

Proposed mitigation strategies for *Aphanomyces astaci* epizootics in astacid populations include: (a) prohibiting the use of fishing gear previously employed in infected waters to prevent mechanical transmission, (b) prompt removal of moribund and deceased hosts from shared water bodies upon initial suspicion of infection to decrease zoospore propagation, and (c) routine sentinel monitoring of aquaculture-designated waters by placing uninfected cages at multiple locations for 2–3-month intervals to survey for *Aphanomyces astaci* establishment via subsequent molecular diagnostics, necessitating a mandatory 5-year farming moratorium in positive waters [34,73].

Other pathogenic oomycetes, including the opportunistic *Saprolegnia* spp., have additionally been documented infecting Astacidea. While *Saprolegnia* spp. predominantly invade dead eggs and larvae, damaged cuticle in living hosts can facilitate rapid transmission and elevated mortality rates [74]. A native oomycete, *Saprolegnia parasitica*, is known as a fish parasite; it can infect crayfish at high stocking densities and is especially dangerous in the periods of mass molting marked by transient cuticular fragility [73].

Several fungal pathogens within the Ascomycota class Sordariomycetes and genus *Fusarium* have additionally been reported to infect astacid hosts, though species-level identifications are often lacking [69]. As filamentous soil saprobes and phytopathogens, *Fusarium* spp. mycelia can elicit disease upon opportunistic invasion. Infested astacids exhibit delayed ecdysis and excessive melanized encapsulation responses within the gill lamellae—termed “black gill disease.” Histological analyses reveal *Fusarium* hyphae are contained within dense hemocytic nodules, with encapsulation extent and hemocyte melanization increasing with disease progression. Production of exotoxins by the fungi may be responsible for its lethality to crayfish [69].

Recommended strategies to mitigate saprolegniasis and mycotic diseases in astaciculture include: (a) rearing monocultures without fish to preclude pathogen introduction while restricting fence breaches by wild vectors, (b) drying and disinfecting ponds between growth cycles, (c) maintaining optimal stocking densities, (d) banning nets that may abrade the cuticle, (e) preventing agricultural runoff containing spores, and (f) applying heat treatment to feed grain to eliminate fungal contaminants and inhibit transmission [34,73].

### 4.2. Cannibalism

Previous research conducted by European specialists has revealed that high mortality rates are a common occurrence in crayfish farming [37]. Intraspecific aggression and cannibalism may significantly contribute to such declines, especially when freshly molted animals with transient cuticular weaknesses are victims of conspecific attacks.

Borisov and Tertitskaya [75] examined the effects of different artificial habitat substrates on injury, mortality, and distribution patterns in a population of 1-year-old *Pontastacus leptodactylus* reared at high densities in an experimental 280-L tank with a total bottom area of 0.94 m^2^, divided into three parts, each containing different substrates: (a) bricks with holes, 23 mm in diameter; (b) a mixture of sand, fine gravel, coarse gravel, *Dreissena polymorpha* druzes, and shells, simulating the rocky bottom of a natural water body with numerous micro-shelters; and (c) plastic tangled threads simulating a complex habitat of aquatic plants [75]. Over the 117-day study, the plastic tangled threads yielded the highest survival rate (85%), though with the most frequent limb injuries (46% affected, 1.04 injuries per crayfish). The brick substrates showed an intermediate survival rate (50%) but the least trauma per individual (31% affected, 0.5 injuries per crayfish). Across treatments, newly molted astacids sustained the majority of documented attacks. Observations revealed a tendency for crayfish to be evenly distributed among plastic threads. In “natural” substrates, crayfish dug little holes under large stones and used empty *Dreissena* shells as shelters. Crayfish were observed to occupy small holes in and small cavities under bricks. It was recommended to use plastic tangled threads along with mink shelters to reduce cannibalism in *Pontastacus leptodactylus* [75]. For comparison, a recent Turkish study has also shown different survival rates of narrow-clawed crayfish on different substrates, with the small-stone treatment group having the highest rate of 77.3%, followed by artificial ropes (65.3%), and no substrate groups (41.3%), during a 90 d culture period [76]. Cannibalism in crayfish under controlled conditions has been shown to be modulated by other factors, such as photoperiod and feeding density. In particular, Farhadi et al. [77] showed that narrow-clawed crayfish reared under a short photoperiod and at high density had higher cannibalism rates than those under other treatments due to more frequent social interactions and higher levels of molting events during dark hours; they concluded that the 18 h light and 6 h dark photoperiod is optimal for crayfish cultivation.

For comparison, the mortality rate of juvenile *Astacus astacus* ranges from 40% to 70% [78] and can potentially reach 95% or higher [79]. The primary factor contributing to such high mortality rates is antagonistic behavior, which leads to cannibalism. Taugbøl and Skurdal [80] noted high mortality during periods of intense molting activity. Though individual rearing systems could theoretically eliminate cannibalistic losses, no economically viable options currently exist.

### 4.3. Domestication

The long-term viability of astaciculture based on native species relies on multiple factors including substantial existing consumer demand and market value for crayfish products, availability of suitable habitat and robust wild founder populations for periodic stock enhancement, and site-specific technological developments enabling sustainable production under local environmental conditions [33].

Several biological traits make native European astacids such as *Astacus astacus* and *Pontastacus leptodactylus* suitable candidates for domestication and commercial production, including their high nutritional value, strong consumer demand and market prices for resultant seafood products, their ability to survive, grow, and reproduce in artificial aquatic habitats given proper environmental conditions, and their low feed costs. However, challenges hindering effective cultivation include reduced resistance to infection (especially to *Aphanomyces astaci*), high sensitivity to water quality, relatively slow growth rate, with crayfish reaching commercial size at ages 2–3 years, regular molting events leading to mortality, and aggressive behavior leading to cannibalism, necessitating relatively low stocking densities. Additionally, some crayfish species exhibit low adaptability to environmental conditions outside their native regions and therefore are best cultivated in water bodies located within their area of distribution. Ponds for broodstock populations necessitate high volumes of high-quality fresh water and specific design features allowing rapid dumping and refilling [33].

A comparative analysis indicates that *Pontastacus leptodactylus* exhibits a higher potential for domestication in comparison to *Astacus astacus* (Table 4).

Nevertheless, a number of overarching issues continue to impede the extensive commercial expansion of astaciculture in Russia. These include a lack of adequate financial and policy support mechanisms, a dearth of specialized technical expertise among producers, and an inability to continuously refine technological components and system parameters to align with the unique biological requirements and environmental sensitivities of target species.

### 4.4. Crayfish Handling

Stress significantly affects crayfish survival and quality post-harvest, and avoiding it is critical during handling and transportation [82]. Freshwater crayfish can survive out of water for extended periods, which offers flexibility in transport and storage [83]. However, to minimize stress and mortality, crayfish must be handled gently and briefly, kept moist to preserve gill functionality, and maintained in cool environments to reduce evaporation and lower metabolic rates [84].

**Figure 5:**
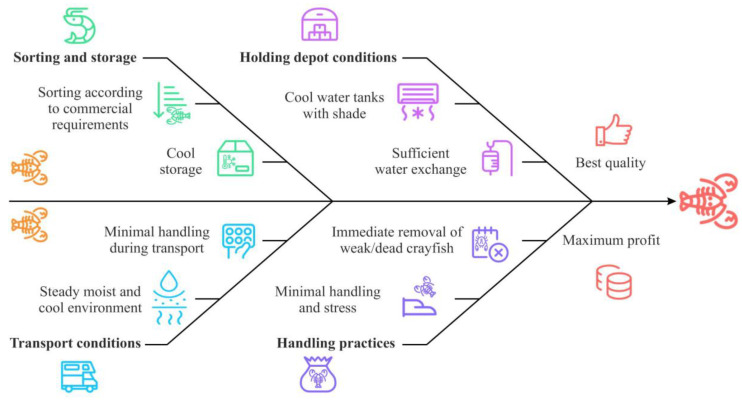
Post-harvest handling of freshwater crayfish.

Throughout the transport chain, crayfish require a moist and cool environment to prevent gill desiccation and maintain gas exchange [85]. Unlike those of fish, crayfish gill filaments function normally out of water as long as they remain moist, while cooler conditions prevent stress-related losses by reducing metabolic activity [82]. A commonly used method involves foam boxes with cooling units at the bottom, separated by a plastic or rubber grid to avoid frostbite. Crayfish are placed in plastic bags to maintain moisture and prevent evaporation and direct sunlight exposure [86]. This approach has been shown to minimize mortality and ensure cool, manageable crayfish at the destination [82]. During land transport, crayfish must be protected from heat, light, and excessive evaporation. Covered trailers or trucks equipped with cooling systems are ideal for maintaining optimal conditions. Containers should also be secured to prevent unnecessary movement, as excess shaking can further stress the crayfish [82]. The main principles during post-harvest handling are summarized in Figure 5.

Future developments may focus on enhanced container designs, automated temperature and humidity control, and clear industry-wide welfare guidelines to ensure the ethical handling of crayfish from point of harvest to market. Ethical standards in crayfish handling should focus on minimizing unnecessary harm, ensuring humane treatment, and maintaining environmental conditions that align with their biological needs during post-harvest processes and transport [87].

## 5. Conclusions

Currently, the crayfish fauna native to Russia includes seven species, among which *Astacus astacus*, *Pontastacus leptodactylus*, and *Pontastacus cubanicus* demonstrate the greatest potential for aquaculture exploitation. To satisfy the growing demand for crayfish products and rehabilitate depleted natural populations, Russian specialists have developed specialized aquaculture techniques, including extensive and semi-intensive pond cultivation and rearing in recirculating aquaculture systems. Innovative strategies for ensuring stable crayfish production have been developed and documented, emphasizing the critical importance of optimal feeding practices and conditions, as well as suitable substrates, for the successful advancement of the industry. However, the growth of the domestic crayfish industry is impeded by insufficient financial and policy support, overfishing, challenges associated with cannibalistic behavior, and the high sensitivity of crayfish to pollutants and diseases.

## Figures and Tables

**Table 1:** Characterization of water bodies in the forest zone of the European part of Russia suitable for crayfish extensive cultivation (modified from [18,32]).

Parameter	Noble Crayfish	Narrow-Clawed Crayfish
Location and hydrology	Clear glacial lakes and the upper sections of their flowing rivers (north-western part of Russia, Leningrad Oblast)	Bays of plain rivers, lakes, and quarries with clean water (Upper and Middle Volga basin)
Catchment area conditions	Predominance of non-swamp forests and meadows; absence of pollution sources and low recreation activity	Predominance of non-swamp forests and meadows; absence of pollution sources and low recreation activity
Pond area, ha	10–100	100–1000
Pond depth, m	4 m	4 m
Yearly water exchange, km^3^ For deep water bodies For intermediate water bodies For shallow water bodies	<0.25 0.25–1 >4–16	<0.25 1–4 >4–16
Averaged water temperatures in summer, °C	10–15	15–20
Degree-days with t > 10 °C	1000–2000	>2000
Area of potential crayfish colonization, %	≥30	≥30
Area occupied by aquatic plants, %	≥30	<25
Zooplankton biomass, g m^−3^	<2.55	n.d.
Zoobenthos biomass, g m^−2^	>10	n.d.

Note. n.d.—no data.

**Table 2:** Optimal water quality parameters for crayfish ponds under extensive culture (modified from [31,32,35]).

Parameter	Noble Crayfish	Narrow-Clawed Crayfish
Maximum water temperature, °C	<24	<26
Water transparency established with a Secchi disc, m	4–8	>1–2
Dissolved oxygen, mg L^−1^	>6–7	>5–6
pH	7.5–8.3	6.8–8.8
Ca^2+^ + HCO_3_^−^, %	90	76
Ca^2+^, mg L^−1^	42.2–48.8	32.0–64.2
HCO_3_^−^, mg L^−1^	171.4–210.4	113.0–190.0
Fe, mg L^−1^	<0.5	<0.5
NH_4_^−^, mg L^−1^	<0.06	<0.1
NO_3_^−^, mg L^−1^	<0.03	<0.03
Permanganate oxidizability, mgO L^−1^	<10	<15
Bacterial abundance, million cells L^−1^	<1.5	<2
Saprophytic bacteria, thousand cells L^−1^	<0.5	–
Ratio of cocci to rod-shaped bacteria	–	1:0.9
*Escherichia coli*, thousand cells L^−1^	<10	–
Zooplankton biomass, g m^−3^	4–7	1.5–5
Zoobenthos biomass, g m^−2^	9–36	3–4
Aquatic flora	Charophyceae	Potamogetonaceae, Ceratophyllaceae, Nymphaeaceae
Aquatic plant area, %	>20	15–20

**Table 3:** Rations for different-aged crayfish *Pontastacus cubanicus* (modified from [46]). w.w.—wet weight.

Stage/Age	Mean Crayfish Weight, mg	Natural Feeds		Formulated Feeds	
		mg w.w.	% from Crayfish w.w.	mg w.w.	% from Crayfish w.w.
Larvae	34.65	13.39	38.50	4.5	13.0
Juvenile 0+	121.30	27.22	22.44	9.1	7.5
Juvenile 24d	238.20	30.09	16.41	13.0	5.47
Juvenile 40d	591	78.90	13.35	26.3	3.4
Juvenile 60d	1506	122.14	8.11	40.7	2.7
Juvenile 90d	2905	184.42	6.40	61.47	2.1
Juvenile 120d	5080	275.59	5.43	91.86	1.81
Juvenile 1+	7710	457.97	5.94	152.65	1.98
Juvenile 1+	17820	559.55	3.14	186.51	1.04
Juvenile 1+	31992	642.29	2.01	214.1	0.67

**Table 4:** Domestication potential of crayfish species in Russian waters. Domestication requirements follow Webber and Riordan [81]. Scores: 2—close correspondence, 1—partial correspondence, 0—inconsistency (modified from [33]).

Attribute	Noble Crayfish	Narrow-Clawed Crayfish
High nutrition quality and prices for products	2	1
High adaptability to artificial conditions	2	2
High ability to reproduce under artificial conditions	2	2
Rapid growth rate	0	1
Low aggressiveness under joint cultivation	1	0
Resistance to disease	0	1
High survival and yield rate	1	2
High tolerance to environmental disturbances	0	1
Diet plasticity and low feed costs	2	2
Mean score	1.11	1.33

## Data Availability

Not applicable.
